# Tackling the primary healthcare workforce crisis: time to talk about health systems and governance—a comparative assessment of nine countries in the WHO European region

**DOI:** 10.1186/s12960-024-00965-2

**Published:** 2024-12-31

**Authors:** Ellen Kuhlmann, Michelle Falkenbach, Monica Georgina Brînzac, Tiago Correia, Maria Panagioti, Bernd Rechel, Anna Sagan, Milena Santric-Milicevic, Marius-Ionuț Ungureanu, Iris Wallenburg, Viola Burau

**Affiliations:** 1 Institute for Economics, Labour and Culture, Goethe-University Frankfurt, Eschersheimer Landstraße 121, 60322 Frankfurt, Germany; 2 WHO Collaborating Center for Health Workforce Policies and Planning, Instituto de Higiene e Medicina Tropical, IHMT, Universidade Nova de Lisboa, UNL, Lisbon, Portugal; 3 European Observatory on Health Systems and Policies, Brussels, Belgium; 4 Department of Health Management and Policy, University of Michigan, Ann Arbor, MI USA; 5 Department of Public Health, Faculty of Political, Administrative and Communication Sciences, Babeș-Bolyai University, Cluj-Napoca, Romania; 6 Center for Health Workforce Research and Policy, Faculty of Political, Administrative and Communication Sciences, Babeș-Bolyai University, Cluj-Napoca, Romania; 7 Associate Laboratory in Translation and Innovation Towards Global Health, LA-REAL, Global Health and Tropical Medicine, GHTM, Instituto de Higiene e Medicina Tropical, IHMT, Universidade Nova de Lisboa, UNL, Lisbon, Portugal; 8 NIHR School for Primary Care Research, Division of Population Health, Health Services Research & Primary Care, University of Manchester, Manchester, UK; 9 European Observatory on Health Systems and Policies, London School of Hygiene & Tropical Medicine, London, UK; 10 WHO European Centre for Primary Health Care, Almaty, Kazakhstan; 11 Laboratory for Strengthening Capacity and Performance of Health System and Workforce for Health Equity, Institute of Social Medicine, Faculty of Medicine, University of Belgrade, Belgrade, Serbia; 12 Erasmus School of Health Policy & Management, Erasmus University Rotterdam, Rotterdam, The Netherlands; 13 Department of Public Health, University of Aarhus, Aarhus, Denmark

**Keywords:** Primary healthcare, Healthcare workforce crisis, Health workforce policy, Health systems, Governance, Policy implementation, Comparative assessment, European region

## Abstract

**Background:**

Primary healthcare has emerged as a powerful global concept, but little attention has been directed towards the pivotal role of the healthcare workforce and the diverse institutional setting in which they work. This study aims to bridge the gap between the primary healthcare policy and the ongoing healthcare workforce crisis debate by introducing a health system and governance approach to identify capacities that may help respond effectively to the HCWF crisis in health system contexts.

**Methods:**

A qualitative comparative methodology was employed, and a rapid assessment of the primary healthcare workforce was conducted across nine countries: Denmark, Germany, Kazakhstan, Netherlands, Portugal, Romania, Serbia, Switzerland, and the United Kingdom/ England.

**Results:**

Our findings reveal both convergence and pronounced diversity across the healthcare systems, with none fully aligning with the ideal attributes of primary healthcare suggested by WHO. However, across all categories, Denmark, the Netherlands, and to a lesser extent Kazakhstan, depict closer alignment to this model than the other countries. Workforce composition and skill-mix vary strongly, while disparities persist in education and data availability, particularly within Social Health Insurance systems. Policy responses and interventions span governance, organisational, and professional realms, although with weaknesses in the implementation of policies and a systematic lack of data and evaluation.

**Conclusions:**

Aligning primary healthcare and workforce considerations within the broader health system context may help move the debate forward and build governance capacities to improve resilience in both areas.

**Supplementary Information:**

The online version contains supplementary material available at 10.1186/s12960-024-00965-2.

## Background

Primary healthcare (PHC) has emerged as a powerful global concept aiming to improve health outcomes, enhancing health system efficiency, resilience, and equity. It has inspired many policy changes, advocating for its prioritisation on the global health agenda [1–12]. Within the European region, PHC has been the focal point of discussions in various meetings of the World Health Organisation (WHO) signalling a collective commitment to action [13, 14]. These discussions have been reinforced by advancements in data and research including on the healthcare workforce (HCWF) [6, 15–20], providing evidence of PHC’s efficiency, specifically during crisis conditions [9, 21–25].

PHC ‘stands as the principal interface between the health system and communities’ [26, website], embodying the convergence of public health and medical care [7, 27]. Defined as a whole-of-society approach, it is based on multisectoral and inclusive policies supporting ‘first-contact, accessible, continued, comprehensive and coordinated patient-focused care’ [7, see also 4, 26]. The PHC workforce ‘includes all occupations engaged in health promotion, disease prevention, treatment, rehabilitation, and palliative care services, as well as addressing the social determinants of health inclusive of caregivers and volunteers’ [28]. Multidisciplinary teams comprising of various healthcare professionals play a key role in providing effective PHC services [19, 28].

Although significant progress has been made in advancing PHC [4, 8, 13, 29], some gaps persist, particularly surrounding the integration of PHC and HCWF debates and its prioritisation within national health agendas [30]. The disconnect between PHC implementation and HCWF exasperates the workforce crisis and obstructs effective service provision. Recent WHO efforts [26] have underscored the challenges in aligning the global PHC-oriented model with national health systems [13], highlighting the need for tailored approaches to fulfil diverse needs and contexts. Others have addressed the ‘layered’ nature of the PHC workforce crisis and emphasised that the ‘causes at the heart of such crisis, and their patterns and implications differ across the very diverse European region’ [30]. Longing for a broad and inclusive ‘one-size-fits-all’ PHC model may risk obscuring these layers and diverse conditions and institutional prerequisites, including powerful professional stakeholders and interests, necessary for effective policy recommendations [12, 20, 31–34].

PHC’s far-reaching promises of universal health coverage (UHC) [4, 29, 35] and equity [4, 29, 36] strengthen the appeal of uniform concepts silencing the critical debate and eventual emerging controversies pertaining to strategies, actors and future directions [12, 20, 32, 37, 38]. Crucially, governance largely remains a black box, hiding the systematic analysis of transformative capacities and making PHC workforce governance poorly prepared for the implementation challenges embedded in politics, policy, and the powers of stakeholders within health systems.

The HCWF crisis poses significant challenges to PHC provision, yet the strategic importance of the HCWF as the backbone of every PHC system and the transformative role of HCWs remain understudied and underappreciated. HCWs are largely reduced to ‘operational’ dimensions [6, Fig. 1], ignoring their role as agents and professional policy actors [39]. This oversight inflames the burden on individual HCWs [40–44] hindering recruitment, retention, and workforce resilience [45, 46]. Addressing these challenges will require an increase in knowledge exchange between countries looking for evidence-based good practice strategies.

Despite the importance of comparative PHC workforce research, it remains underdeveloped with broad and diverse definitions (for an overview, see [26, Table 3.1]) and recommendations [6, 19, 28, 47] hindering empirical operationalisation. Key areas, such as the HCWF and composition and functioning of multidisciplinary teams [48, 49] and the role of community health workers [22, 28, 50], require further examination to effectively inform policy and research moving forward. The lack of PHC-disaggregated workforce data and monitoring systems in most countries, except for physicians [16, 18] further complicates efforts to assess PHC performance and identify areas for improvement [48, 51, 52].

Our comparative assessment aims to bridge the gap between the PHC policy debate and the HCWF crisis debate by introducing a health system and governance approach [2, 3, 8] to contribute new knowledge and identify capacities that could support effective responses to the HCWF crisis within different health system contexts.

## Methods

We employed a qualitative comparative methodology, which is explorative and informed by health systems and governance theories [11, 53–55]. Drawing upon insights from previous research [39, 41], this study aimed to investigate the complex interplay between PHC workforce dynamics and health system governance within the WHO European region [2, 3, 8]. A rapid assessment of the PHC workforce was performed based on a case study design and expert information.

### The country sample

The country sample includes nine countries: Denmark, Germany, Kazakhstan, Netherlands, Portugal (excluding the Azores and Madeira), Romania, Serbia, Switzerland, and the United Kingdom with a focus on England. The selection aimed to capture a diverse array of healthcare systems including the National Health Service (NHS)/Beveridge, Social Health Insurance (SHI)/Bismarckian, and emergent (post-communist) SHI systems in Central Eastern and Eastern Europe, while also accounting for diversity within ideal-type systems. By going beyond typological categorisations, this study embraced diversity allowing for the examination of various contextual factors to gain deeper insights into the PHC workforce crisis and inform actionable interventions [39, 41, 53, 54].

Across countries, various terminologies exist to describe PHC and its practitioners. In this study, we use PHC, ambulatory general care and family care, as well as GP and family physician synonymously. A further relevant term is corporatism, generally defined as a model of governance where interest groups exercise power in political decision-making; in the health sector professional actors and provider organisations play a key role, especially ‘doctors are an integral part of health governance’ [11, p. 162].

Table 1 provides an overview of the country sample, considering different types of healthcare systems, PHC models, labour market conditions and workforce compositions, and geographical variation [16, 56, (Denmark) 57, (Germany) 58, (Kazakhstan) 59–62, (Netherlands) 63, (Portugal) 64, (Romania) 65, (Serbia) 66–68, (Switzerland) 69, (United Kingdom/England) 70].

**Table 1:** Country sample: health systems and healthcare workforce figures

Categories	Denmark	Portugal	UK	Germany	Netherlands	Switzerland	Kazakhstan	Romania	Serbia
Health system/ governance	NHS, public & some professional corporatism; decentralised	NHS, public & professional corporatism; partly decentralised	NHS, some corporatism; centralised but devolution	SHI with joint self-governance & corporatism; decentralised	SHI with regulated competition & corporatism; centralised	SHI with managed competition & corporatism; decentralised	SHI with strong state regulation, very little corporatism; centralised	SHI with some state regulation & corporatism; partly decentralised	SHI with state regulation & little corporatism; centralised
Total health expenditure, %GDP*	9.5	10.6	11.3	12.7	10.2	11.3	3.79^#^	6.27^#^	8.73^#^
Health & social work, % total civilian employment*	18.22	8.71	12.93	13.94	16.12	14.46	6.4^Ω^	4.89	7.03^∫^
Total health & social employment, density*	94.76	41.67	62.21	74.37	89.79	84.51	n/a	22.18	24.35^∫^
Physician density*	4.38	n/a	3.18	4.53	3.9	4.44	2.98^§^	3.51	2.70^§^
Physicians aged ≥ 55, % of all physicians*	29	n/a	14	45	24	37	n/a	20	n/a
GP density*	0.80	2. 97	0.81	1.03	1.83	1.14	n/a	0.79	0.60^≠^
GPs, % of all physicians*	18.17	53.05	25.41	22.85	46.88	25.8	n/a	25.58	19.37^≠^
Nurse density*	10.24	n/a	8.66	12.3	11.38	18.39	6.51^§^	7.99	5.79^§^

Sources: authors’ own table, updated and amended [41]

*All countries, except Serbia and Kazakhstan, 2022 or nearest year; workforce data refer to: practising physicians; head counts of physicians/nurses per 1000 population[16]

^#^Kazakhstan, Romania, and Serbia [56]

^Ω^Kazakhstan, calculated from a survey 2021 [62]

^§^Kazakhstan and Serbia: physician density, nurse density [17, p. 133, p. 167]

^∫^Serbia: [67, p. 68]

^≠^Serbia: [68, p. 34]

*n/a*  data not available or dated

### Data collection and analysis

Data collection primarily relied on inputs from country experts and secondary sources, such as public statistics, policy documents, and published research (for details, see supplementary material 1). We developed an assessment tool synthesised from the PHC [3, 6, 7, 24, 26, 27] and HCWF literature [17, 41, 54, 55, 71–73]; relevant items were identified and operationalised into a semi-structured matrix (supplementary material 2). This matrix served as a framework for gathering country specific information focusing on recent developments while considering broader institutional and workforce conditions.

A step-by-step explorative team-based approach guided the iterative analysis process. The lead authors produced summaries of the country cases that were then reviewed and revised in consultation with country experts. The iterative process continued until sufficient coherence and clarity were achieved for each country case. From this explorative analysis, three categories (comprising sub-categories) emerged as a framework for a rapid comparative assessment: PHC systems (Table 2), PHC workforce (Table 3), and PHC workforce action taken (Table 4). System types served to structure and proved a framework for organising the complex qualitative data, enabling an exploration of trends and variations across countries.

**Table 2:** Primary healthcare systems

Primary healthcare systems (1) Governance, (2) finance and (3) provision		
NHS systems	Denmark	1. Participatory governance (multi-level), strongly decentralised and community-centred (municipalities), with public corporatism, and inclusion of different HCWs and major professional interest groups. The National Board of Health is responsible for physicians and nurses, while the Ministry of Children and Schools takes on the responsibility for social and healthcare assistants. Professional associations have the status of a trade union and negotiate salaries. There is strong transsectoral coordination because of the regional health agreements and the sub-regional networks that offer a framework for coordinating PHC across GP offices, municipalities and hospitals 2. Financed through national and local-level taxes and capitation is the main reimbursement model defined by framework contracts at regional level 3. Provision flows through a regional/municipal health system with UHC free at point of care. PHC includes: general practices (independent private businesses contracted by regions) and municipalities (local governments). GPs have a gate-keeping role. They offer: general medical diagnosis and treatment, and prevention services for all groups (including basic gynaecology/maternity care); however, dental care is excluded. Municipalities offer a broad range of services, including: health promotion, rehabilitation, school nursing and health visitor services, community-based mental health services, intermediate care, and elderly care services
	Portugal	1. Hierarchical governance with multi-level structures, co-existence of public and professional corporatism, partly decentralised. Decentralisation has been strengthened and new efforts are underway to reinforce governing capacities of municipalities, yet coherent transsectoral coordination is lacking. The state ensures financial governance and regulates the labour market, the salaries of HCWs as public servants, and graduate education, even in private universities. Physicians’ and Nurses’ Associations (*Orden*) are strong corporatist actors with self-governing powers. The state regulation of HCW salaries allows for some operational flexibility through combining different payment modes according to the type and need of the organisational setting 2. Financed via national taxes with different financing mechanisms according to the types of units providing PHC. Family Health Units: payment systems for staff varies according to the development model of the Family Health Units, with collective and individual incentives and customised health units; a combination of variable and performance-based remuneration can be used to motivate retention 3. Provision through the NHS with UHC and access free at point of care. PHC is provided by mainly multi-professional teams in Health Center Clusters each of them enrolling between 50,000 and 200,000 people; GPs have a strong gate-keeping role; HCWs are public sector employees. A new policy aims to merge Health Center Clusters and hospitals. PHC includes: medical diagnosis and treatment for all age groups (including maternal care and some basic dental care), health promotion/illness prevention, and some public health tasks
	UK/ England	1. Hierarchical governance with weak corporatism. The contract between NHS England (main regulatory body) and GPs forms the pillar of PHC, while the Clinical Commissioning Groups regulate the local level provision. Workforce governance is overseen by NHS England, while Health Education England is accountable for education, and NHS Employers and the Department of Health and Social Care take care of contractual regulations. Professional bodies (General Medical Council, Nursing and Midwifery Council) have self-regulatory professional rights, but are overseen by the NHS. Comprehensive coherent coordination mechanisms are missing 2. Financed via national taxes with small shares of private funding. GPs are reimbursed based on a capitation system with some entrepreneurial and pay-for-performance elements 3. Provision with UHC and access free at point of care, based on framework agreements and a strong gatekeeping role of GPs. Organisational settings and the substance of service provision vary significantly due to devolution politics. The NHS England establishes large Primary Care Networks, covering populations of around 50,000. GPs are mostly NHS employees, but new contracts open the door for private practice and entrepreneurship. PHC includes: general practice (including basic gynaecology and maternity care), community pharmacy and some basic dental and optometry services for all age groups. Primary Care Networks offer: preventative and health promotion services, vaccinations, prescriptions, and referrals to other specialised and social services
Established SHI systems	Germany	1. Participatory governance with strong corporatism, decentralisation, sectoral fragmentation, and organisational diversity. SHI Insurance Funds and SHI Physician Associations form the self-governing and self-administering bodies of the SHI system; they jointly negotiate contract frameworks, reimbursement schemes and budgets. Stronger marketisation and privatisation have opened the door for new forms of contract agreements, that weaken the governing powers of the key SHI stakeholders. Comprehensive coordination mechanisms are lacking. SHI Physician Associations have statutory rights, while other HCW groups are external policy players. Multi-level and transsectoral governance and coordination are weak 2. Financed through SHI-based mandatory insurance with some tax-funding and little out-of-pocket payments. Reimbursement is negotiated within the SHI joint self-regulatory bodies. SHI Insurance Funds and SHI Physicians agree on a joint budget for ambulatory care, which SHI Physician Associations subsequently allocate between PHC and specialist physicians. Reimbursement of PHC physicians is mainly based on fee-for-service with some mixed models and diversity 3. Provision through self-employed office-based physicians in single- or group practice based on UHC and free access at point of care with no mandatory gatekeeping. The dominant model of collective contracting between SHI funds and SHI Physicians is expanded towards selective contracting (between SHI funds and physicians) aiming to foster integrated care and organisational diversity, including large centres run by private business companies. Provision includes: general medical (family medicine) ambulatory diagnostic and treatment for all groups, some preventative services, some health promotion, GP home visits and coordination of care. Larger PHC Centres may provide a wider range of services. Dental care, eye care, gynaecology, and elder care/nursing are not part of PHC
	Netherlands	1. Participatory governance with national regulation, regulated competition and strong corporatism. The Dutch Health Care Authority is the main regulatory body of insurance companies and providers, controlling the coordination of services and financial arrangements of the workforce. Health insurances have legal responsibility to purchase high-quality care services. Efforts to strengthen community-centred governance and provision of PHC are underway but implementation is currently not clear. Some coordination mechanisms have been established. Corporatism is multi-professional including physicians, nurses, midwives, physiotherapists, pharmacists, and other HCW groups 2. Financed through mandatory SHI (private insurance with public regulation) with relevant market and out-of-pocket payment (for some services). Reimbursement of GPs is characterised by mixed models (fee-for-service, capitation, incentives) and diversity 3. Provision through self-employed office-based physicians with increasing organisational diversity, i.e. midwives play a dominant role in maternity care; single GP practices are replaced by group practices and PHC Centres including multi-professional provider models with midwives and physiotherapists are independent professional groups with direct patient access. Provision is based on UHC and mostly (some exceptions) free access and an increasingly strong gate-keeping role of GPs. Provision includes: ambulatory diagnostic and treatment for all groups, midwifery/maternity care, physiotherapy, (non-specialised) mental health care, some preventative services and health promotion, care at home/community-based care, and coordination of care. Larger PHC Centres may provide a wider range of services. Dental care, eye care, and elder care/nursing services are not part of PHC
	Switzerland	1. Participatory governance and strong corporatism, strongly decentralised with weak governance at the national level. Each of the 26 cantons is responsible for governing and securing healthcare provision for their populations, including financial issues, based on a joint regulatory framework. The accreditation of GPs is, however, regulated at the federal level. Key professional stakeholders include the Swiss Medical Association, Conference of Regional Health Directors, and cantonal/regional GP Associations with some (still weak) involvement of community authorities. Other important players are Health Maintenance Organisations (HMOs) that may set up direct contract agreements with providers and weaken existing governance frameworks. Coordination mechanisms are generally weak, except in the HMOs 2. Financed through mandatory SHI at Canton level. Highly diverse contract models with provider fee-for-service reimbursement schemes and a high share of out-of-pocket payments (approx. 66%) 3. Provision is strongly physician-centred and provided by GPs in self-employed single- and group practices supported by medical assistants. Provision is based on UHC with no mandatory gate-keeping, but HMO’s may establish integrated contracts with gatekeeping mechanisms. Provision includes: generalists ambulatory care diagnostic and treatment for all groups, vaccination and some preventative and health promotion services, home visits and coordination of care provided by other providers, and a few basic gynaecology services. Larger PHC Centres and HMOs may provide a wider range of services, making provision more diverse. Dental care and elder care/nursing services are not part of PHC
Emergent SHI systems in Central Eastern/ Eastern Europe	Kazakhstan	1. Hierarchical centralised governance with the Ministry of Health as key regulatory body, operationalised regionally through 17 Oblast Health Departments, including accountability for adequate staffing levels, education, and social support plans in rural areas. Operational governance is more diverse, including budget autonomy of most state-based organisations. Corporatism is very weak. Some transformations are underway (multi-professional and community approaches) and coordination has improved, but implementation is highly diverse and information is lacking. Corporatism is weak but increased. However, but key associations (Association of Family Physicians, Associations of Nurses, National Association on Primary Health Care, and Social Worker Alliance) are not part of the governing bodies 2. Financed via SHI with a mix of taxes (about 2/3) and high out-of-pocket/co-payments (about 1/3). PHC providers are salaried Oblast employees; self-employed providers/private business are marginal. Remuneration is mainly based on capitation, universal for the whole country, and some additional pay-for-performance to incentivise referrals to specialists 3. Provision is based on a large and diverse network of PHC facilities that strongly vary between rural and urban regions. A gatekeeping system co-exists with some payment models that incentivise specialised care. Organisational settings are highly diverse and dependent on geographical conditions; in urban areas PHC is provided in multi-speciality and specialised policlinics; in more rural areas by smaller teams, including physician assistants (Feldshers) and midwives as the smallest organisational unit in rural areas; remote areas may be supported by mobile solutions including equipped PHC buses and two trains. PHC comprises of a very wide range of services, including: medical diagnosis and treatment for all groups, some psychological and social support services, coordination with the communities. The share of services provided by specialised physicians is high in PHC
	Romania	1. Hierarchical centralised governance with some participatory governance with Insurance Funds and some corporatism. The Ministry of Health, through its General Directorate for Healthcare and the National Health Insurance House, is legally responsible for PHC, operationalised at the local level through district public health authorities and district health insurance houses. However, the framework contract for PHC provision is negotiated between the College of Physicians and the National Health Insurance House. The College of Physician and the Order of Generalist Nurses, Midwives, and Nurses are the main professional stakeholders, responsible for overseeing professional issues and collaborating with government authorities. Coordination mechanisms are generally weak 2. Financing is primarily based on a mandatory SHI system with some state and local budgets and relevant out-of-pocket payments. Remuneration is based on fee-for-service and per capita payments; salaries are market-based 3. Provision is based on a nationwide network of self-employed office-based GPs working in single- and group-practices with nurses and administrative staff and having a gatekeeping role. Provision is based on UHC and regulated through a Framework Contract between the National Health Insurance House and private office-based physicians, including a basic benefit package for the insured and a minimum package for the uninsured. Major services include: medical non-urgent diagnostic and treatment services for all groups, a broad range of preventive services and health promotion (including vaccination), some basic gynaecology services and maternity care, and coordination with community health workers or health mediators. Dental care is not part of PHC, but efforts have been increased to include preventive services
	Serbia	1. Hierarchical centralised governance with the Ministry of Health as the key regulatory body, based on a Network Plan of State Health Institutions that coordinates the different stakeholders and providers (PHC Centres, Institutes, pharmacies, etc.) with some corporatism, e.g. SHI Funds are responsible for negotiating salaries and remuneration. Professional corporatism is weak and limited to the associations of physicians, dentists, and pharmacists. A small private segment exists that is market-based and operates outside SHI governance and state control. Coordination is weak, however, this is more advanced in the PHC Network 2. Financing is based on a mandatory SHI system with a small portion being taxed-based and high out-of-pocket payments. Salaries and remuneration of medical PHC providers are negotiated annually with SHI funds, based on a mixed system of performance-based payment and capitation 3. PHC is mainly provided by a network of physicians in large state-owned Centres (mostly PHC Centres, some included in hospitals), some institutes, and several pharmacies. A PHC Centre can be established for at least 10,000 residents at the municipality/city level. Providers in the Network are mostly state employees; pharmacists may be self-employed; a small share of private PHC provision is fully office-based and no public data is available. Provision is based on UHC with limited financial protection/high co-payments and mandatory gatekeeping through GPs and some specialist physicians (occupational medicine, paediatrics, gynaecology, dentistry). Major services include: medical diagnosis and treatment for all groups of the population, prevention and health promotion services, prehospital emergency care, geriatrics/palliative care, dental care, pharmacy services, some maternity care, emergency in-patient care if a hospital is too far away, some transportation services, and epidemiology/public health services

Source: authors’ own table; references, see supplementary material 1

**Table 3:** Primary healthcare workforce

Primary healthcare workforce (1) Composition, (2) education, (3) labour market data/planning		
NHS systems	Denmark	1. Main professions: GPs, nurses, health, and social care assistants/helpers. Some skill-mix, task-shifting and new roles of nurses have been established 2. GPs and nurses are academically trained with mandatory specialisation for GPs and voluntary PHC-related specialisation for nurses (advanced practice nurses, community nurses). Care assistants are trained vocationally 3. Sector-specific labour market data are available for all relevant PHC groups. Comprehensive planning has been established at the regional and/or national levels for all PHC groups
	Portugal	1. Occupational groups depend on the type of PHC. Family health units (largest type) consist of multidisciplinary teams, comprising of specialised physicians (GPs/family medicine), public health physicians, nurses (both generalists and those specialised in community and public health), technical officers in environmental and public health, social workers, physiotherapist, occupational therapists, psychologists and nutritionists, dentists, and clinical secretaries. Task-sharing and shifting remains limited and is only described among physicians and nurses 2. GPs, nurses and other professionals are academically educated with mandatory specialisation requirements for GPs, for all other professions this is voluntary 3. Labour market data are not disaggregated for PHC and professional groups, except for GPs. Major trends include shortages, reinforced by ‘ageing cohorts’. No reliable HCW migration data is available, but a negative migration balance has been predicted and rather stable inflows of foreign-trained/born HCWs are common. Publicly available data is limited, and no systematic monitor and planning has been established
	UK/England	1. The PHC team is multi-professional; the composition depends on organisational setting. In Primary Care Networks, major HCW groups include GPs, nurse practitioners and healthcare assistants, supported by pharmacists, physiotherapists, mental health specialists, community nurses, and social workers. Some skill-mix, task-shifting and new roles of nurses have been established 2. GPs are academically educated with mandatory specialisation. Nurses and other middle-level professional groups are academically trained, and practice nurses and physiotherapists can obtain master’s degrees and specialisations, while healthcare assistants mainly obtain vocational training degrees, diplomas or are trained at the job 3. Labour market figures are available and disaggregated for most PHC groups, showing GPs as the largest group followed by healthcare assistants and practice nurses. A shortage of HCWs has been common for years and worsened for GPs; however, the numbers of other HCW groups (physiotherapists, mental health specialists, social workers) have increased. Health Education England and NHS England collaborate on HCWF planning and have established monitoring systems that include GPs and some other HCWs
Established SHI systems	Germany	1. Main professions: GPs together with some internal medical specialists and paediatricians who opted for Family Care, and medical assistants. Nurses are not typically employed in PHC (some exceptions). Multi-professionalism is more common in larger PHC centres 2. Academic education with mandatory specialisation is required for GPs, but not available for other groups. Nurses and other middle-level professionals are mainly educated in the vocational system and academisation remains weak. Vocational education (3 years) is required for medical assistants; some certificate-based PHC (community nurses) training is available for medical assistants and nurses but rarely taken 3. Labour market figures are not disaggregated for PHC. GP figures may be estimated from public statistics, while the Medical Chambers obtain data for medical assistants (the largest group). Comprehensive PHC workforce monitoring and planning are not established, and SHI Physicians Associations are legally responsible for PHC planning
	Netherlands	1. Main professions: GPs, GP practice nurses, midwives, pharmacists, and physiotherapists. Skill-mix and new roles of nurses and other middle-level professional groups have been established; midwives, pharmacists and physiotherapists are independent providers (direct access) 2. The academic education of physicians is required with mandatory specialisation of GPs. In nursing, academic training co-exists with vocational schools; voluntary specialisation is available for GP practice nurses (comparable to Nurse Practitioners), but not for other groups 3. Labour market figures are not disaggregated for PHC, and the number of physicians can be estimated from GP figures. Comprehensive workforce planning is in place, but it is focused on physicians and does not consider sector-specific issues. A national advisory board, under the control of the Ministry of Health, is legally responsible for the monitoring of a wide range of professions
	Switzerland	1. Main professions: GPs with some paediatricians and internal medicine physicians, and gynaecologists. Medical assistants form the largest group, while nurses are an exception. Task-shifting is generally weak with some skill-mix in physiotherapy followed by a few nurses, dietitians and occupational therapists. New roles and more advanced skill-mix are limited and largely absent 2. Academic education and specialisation of GPs is mandatory; however, no specialisation is available for other HCWs. The academic education of nurses is increasing but co-exists with vocational training. Medical assistants are educated in the vocational system (3 years) 3. Labour market figures are not available for the PHC sector, except for GPs. A systematic monitoring and planning system is missing
Emergent SHI systems in Central Eastern/ Eastern Europe	Kazakhstan	1. Main professions: GPs and specialised physicians, district therapists, paediatricians, PHC nurses, midwives, physician assistants (Feldshers), social workers, and psychologists. The PHC workforce is multi-professional, but the composition varies strongly. A typical PHC team comprises of three PHC nurses per physician along with social workers and psychologists. In rural areas physician assistants and midwives build the core team. Expanded and new roles of nurses and task-shifting/skill-mix have been introduced 2. Education and training for all PHC staff improved significantly, unqualified PHC providers are largely suspended. PHC specialisation is established for physicians and nurses, and some facilities introduced multi-disciplinary training courses 3. Labour market figures show a continuing increase in relevant groups in PHC. The share of GPs in relation to specialists also increased but specialised providers remain dominant. Planning and monitoring operate under the umbrella of the Republican Center for Health Development and are based on the Registry of Medical Workforce, a unified database maintained by the Observatory of Medical Workforce. There is no comprehensive sector-specific data, but it can be estimated for GPs and other predominantly PHC-based groups
	Romania	1. Main professions: GPs, nurses, community nurses, health mediators, school physicians and nurses, and support staff. Community health nurses are a relatively new and small group, contributing to providing care especially to elderly and other vulnerable groups, mostly in rural/remote areas. Very little task-shifting has been established 2. GPs are academically trained with mandatory specialisation, but there is little academisation of nurses and other groups, and no PHC specialisation is available 3. Sector-specific PHC workforce data are not available except for GPs. Planning is modestly developed and hampered by different and insufficiently connected data sources
	Serbia	1. Main professions: GPs (with and without specialisation) and specialised physicians, nurses, and a wide range of multi-professional staff (dentists, pharmacists, psychiatrists, health laboratory technicians, physiotherapists, radiology technicians, social workers, administrative staff). Task-shifting is largely absent 2. Physicians are academically educated with voluntary specialisation for GPs. Nurses are mostly educated in a secondary-school system with some academic training being established, while PHC specialisation is lacking for all groups except physicians 3. Labour market data are disaggregated for PHC providers operating under the Network Plan Centres and public data on other/private providers are lacking. The share of specialised physicians is significantly higher than the number of GPs. Nurses are the largest group with numbers about ten times as high as GPs and specialists. Some centralised workforce planning has been established but hampered by poor and scattered data. The Law on Records in Health Care includes the Network of PHC providers with data collected through the Public Health Institutes, and additional information from the Register of Health Care Providers and Register of Employees

Source: authors’ own table; references, see supplementary material 1

**Table 4:** Primary healthcare workforce action

Primary healthcare workforce action (1) Challenges, (2) policy, (3) implementation, (4) global models		
NHS systems	Denmark	1. Major problems include a shortage of all PHC groups, and regional mismatches with more pronounced shortages in rural areas 2. A PHC reform was introduced and additional funding was made available to increase HCW staffing levels. Efforts are underway to create new models of PHC beyond general practice, thereby blurring the boundaries between hospitals and general practice/out-patient care. These models and related funding efforts are driven by the municipalities that focus on care for elder patients, aiming to avoid hospital admissions 3. A national health reform is pending, but there are a wide range of regional and local efforts to improve recruitment and retention in PHC, reflecting the decentralised PHC organisation. Coherent coordinating of PHC across general practice, municipalities and hospitals has been established 4. The WHO PHC model is recognised but does not provide a systematic basis for policy reform and is not connected to the SDGs
	Portugal	1. Major challenges include: HCWF shortages, geographical imbalances, an increase in private sector employment in areas covered by the NHS, limited data and planning, and the growing dissatisfaction of HCWs. Slow and poor implementation of the PHC reform, and gaps between centralised–decentralised governance are also considered problematic 2. Political rhetoric to protect HCWs is strong but lacking action. Some action has been taken to strengthen the position of nurses through an increase in salaries, and through new tasks and responsibilities but the results vary strongly between organisations 3. There are no interventions to improve recruitment, retention and mental health. Policy implementation is poor and strong drivers for change are missing. In addition, coherent transsectoral and multi-professional coordination is lacking 4. The WHO PHC model does not play a relevant role beyond rhetoric, and the SDGs are largely absent from policy discourse
	UK/England	1. Major challenges are shortage of GPs, nurses and other relevant PHC staff, poor recruitment and retention in rural areas, HCW stress/burnout, and unsustainable levels of international recruitment 2. Policies have been introduced to improve education, recruitment and retention, focusing on increasing HCW numbers through international recruitment, introducing new roles, and strengthening task-shifting, team approaches, and mental health support. The NHS Long Term Workforce Plan 2023 provides significant funding for additional education/training places for physicians, nurses, dentists and other HCWs. The Additional Roles Reimbursement Scheme encourages recruitment and inclusion of additional practitioners (e.g., physiotherapists, pharmacists, paramedics) in PHC teams 3. PHC interventions focus on education, professional development, skill-mix, and some organisational change; yet, coherent coordination mechanisms and governance are lacking. Implementation is poor, structural interventions are generally weak, and organisational and professional action are poorly coordinated. Initial evidence shows that new roles have not been effectively implemented into PHC teams 4. The WHO PHC model and SDGs model have little impact on policy and implementation
Established SHI systems	Germany	1. Major challenges include shortages of physicians and medical assistants coupled with geographical mal-allocation, large retirement waves, strong medical hierarchy, weak academisation of nurses, and an expansion of for-profit companies that weaken public control 2. The PHC workforce is not a policy priority and a systematic strategy for improving recruitment and retention is missing. Policies focus predominately on physicians with the aim of increasing the recruitment of foreign-trained physicians and making PHC more attractive 3. PHC interventions are largely physician-centred, including a comprehensive increase in medical training capacity, quotas with prioritised access to medical education for those planning to work in PHC in rural areas, incentives for GP specialisation and PHC providers (shorter education, some recognition of other qualifications). Multi-professional workforce development is lacking, but organisational changes are present seeing an increase in large private PHC centres with multi-professional provider groups supporting skill-mix and team approaches 4. The WHO PHC model and the SDGs are largely missing from the workforce debate
	Netherlands	1. Major challenges include (regional) shortages of GPs and nurses, increasing complexity of services and user needs that cause overburdened physicians, and for-profit organisations taking over GP practices and enhancing change through organisational restructuring without mandate/public control 2. The PHC workforce is among the key policy priorities. There are new policies aiming to align specialised and GP care and improve transsectoral coordination between hospitals, ambulatory care, elder care/nursing homes 3. PHC interventions include new regulations to force specialised care providers to work in PHC, strengthening a community-based approach, and increasing funding for medical PHC provision. However, strong market and corporatism hamper the governing power of the Ministry of Health, increasing complexity and uncertainty in implementation 4. The WHO PHC model and SDGs are largely missing from public debate and their impact is unclear
	Switzerland	1. Major challenges include shortage of GPs, large retirement waves, high shares of part-time work, growing shortage of Medical Practice Assistants (MFAs), negative attitudes towards integrated care and barriers to implementation, lack of attention for MFAs in PHC reform and new HCWF policies 2. PHC is not considered in the Health Strategy 2030, but recent policies and governance innovation may impact the organisation of care and professional roles. This includes new insurance contracts with family doctors as gate-keepers, an increase in GP group practices, improved skill-mix and new professional roles of nurses, and some task-shifting to physiotherapists (direct access) 3. PHC policy interventions and reform modes focus on organisational change to establish integrated care, larger centres and multi-disciplinary group practices. PHC policy interventions ignore the labour market conditions, are not responding to the HCWF crisis, and efforts are not monitored 4. The WHO PHC model is not explicitly connected to PHC and the same applies to the SDGs
Emergent SHI systems in Central Eastern/ Eastern Europe	Kazakhstan	1. Major problems include shortages of PHC staff including teaching staff, geographical maldistribution, poor retention in rural/remote areas (especially for young professionals), a general lack of medical student interest in PHC, the growing privatisation of medical education with poor regulation and quality control, and the poor and uneven implementation of national skill-mix guidelines 2. PHC and the workforce play an important role in health policy. There is a bundle of policy efforts, including increasing the share of GPs/family medicine in relation to specialist services (improved education, retraining of GPs, specialisation of physicians and nurses, improvement of work conditions, increase in salaries), increasing the attractiveness of working in rural areas (financial benefits; social support/housing, etc.), and establishing a multidisciplinary team-based PHC model that expands biomedical approaches towards a more holistic public heath approach 3. Policy interventions are characterised through multi-level trans-sectoral action, the connection of professional and organisational innovation, and a multi-professional focus on the HCWF and skill-mix/teams. Some pilot projects have been established but an overview of its impact is missing 4. The WHO PHC model is a strong driving force for comprehensive system-based policy and PHC transformations including the human resources. The role of SDGs is less clear
	Romania	1. Major challenges include insufficient staffing levels and shortages of physicians and nurses, especially in rural areas, low career attractiveness of PHC for students and early career professionals, poorly regulated scope of practice, high out-migration of physicians, some overproduction in the education system, and inconsistencies in training policies, funding, and access to resources for PHC staff 2. There are some policy efforts to improve education and working conditions through the provision of financial incentives. In additions there are efforts to foster interdisciplinary collaboration within PHC teams (including between family doctors, community nurses and social workers in rural areas) and policies being introduced to strengthen digitalisation 3. A new Recovery and Resilience Plan pays some attention to recruitment and retention, but sector-specific needs of PHC staff are not addressed. Coherent governance and coordination between corporatist stakeholders are lacking, and implementation is not monitored and hardly predictable 4. The WHO PHC model and the SDGs provide some guidance at national-level policy development
	Serbia	1. Problems are mostly a result of general policy failures and a lack of planning. There is an overproduction of well-educated HCWs (physicians and nurses) with high out-migration, underfunding and understaffing of the public sectors, poor work conditions in PHC, a lack of mental health support, low salaries, geographical and sectoral mismatches with strong PHC staff shortages and large cohorts of HCWs nearing the retirement age 2. A national PHC workforce plan is lacking, and the responsibility is delegated to the operational level of organisations. The Midterm Health Strategy of the Ministry of Health (2022–2025) includes the development of HCWF planning, but without attention to the PHC workforce. Some efforts are underway to improve the situation in future, including the Network Plan, digitalisation of the PHC workforce, and the introduction of a PHC model based on family medicine that is led by WHO 3. Policy interventions are generally poor and largely absent for the PHC workforce 4. The WHO PHC model and SDGs provide some guidance but implementation is poor if not absent

Sources: authors’ own table; references, see supplementary material 1

## Results

### Primary healthcare systems

#### NHS systems

The three different NHS systems show some similarities in funding and organisation, yet differences prevail, most strongly in governance. PHC governance ranges from participatory decentralised multi-level governance with comprehensive coordination mechanisms and community-orientated approach in Denmark to more hierarchical forms with weaker coordination in England and Portugal. The inclusion of corporatist actors and self-governing capacities is strong in Denmark and less so in Portugal, and weakest in England. While funding in NHS countries primarily relies on taxes, reimbursement schemes vary, including capitation, pay-for-performance, and mixed incentives. The provision is based on UHC principles with GPs serving as strong gatekeepers and organisational shifts towards larger centres offering comprehensive services for all users. The range of services is generally broad across the countries, including basic maternity care in all countries, and limited dental care available in Portugal and England.

#### SHI systems

Historically routed in similar institutions, established SHI systems feature SHI Funds and Physician Associations as key stakeholders and are based on participatory governance and strong corporatism. However, differences have increased in all areas over time, particularly amongst governance structures. Levels of centralisation vary across countries with decentralised governance in Germany and Switzerland and centralised structures in the Netherlands. Coordination mechanisms range from advanced in the Netherlands to weaker in Switzerland and governance transformation efforts differ with some community-centred approaches in the Netherlands, but no substantive changes in the other countries. PHC workforce governance mirrors this complexity with multi-professional bodies and decentralised physicians-centred decision-making with weak coordination in Switzerland and Germany and more advanced efforts in the Netherlands. Funding is mainly based on SHI contributions, with Switzerland and the Netherlands featuring higher shares of private insurances and/or out-of-pocket payments. Fee-for-service dominates reimbursement schemes and provision is largely provided through contracted private businesses, with a trend towards larger centres and integrated care models. PHC physicians have a gate-keeping role in the Netherlands, while free provider choice prevails in Germany and Switzerland. The range of provisions is similar amongst the three countries and focuses on general medical care with little prevention and promotion services. Specialised care is separate and builds the second pillar of out-patient/ambulatory care. Dental care is not part of PHC, and maternity care is only included in the Netherlands and predominantly led by midwives.

#### Emergent SHI systems

Emergent SHI systems exhibit hierarchical governance with varying degrees of centralisation, participatory elements, and stakeholder involvement. Romania focuses on professional corporatism, Serbia emphasises SHI funds, while Kazakhstan demonstrates weak involvement of both. Funding models resemble those of established SHI systems, with SHI contributions supplemented by out-of-pocket payments, although private payments are highest in Kazakhstan. Provision is based on national networks and framework agreements with mandatory gatekeeping and diverse provider models, ranging from mainly self-employed practitioners in Romania to larger state-owned centres in Serbia. Kazakhstan shows stark differences between rural and urban regions and its organisational models include mobile solutions and small units as well as large hospital-based centres. PHC in these countries covers a wide range of basic services for all groups, alongside public health, and some specialised services, although the depth of coverage varies. In Serbia and Kazakhstan, PHC serves as an umbrella for both specialised and generalist providers of out-patient care with specialists taking a powerful position, while GPs are the main PHC providers in Romania.

#### Trends across healthcare system types

Overall, although some convergence is discernable, such as more diverse and mixed funding systems and organisational transformations supporting the establishment of larger centres, and increased stakeholder participation in governance, diversity remains pronounced across the PHC systems. None of the countries fully align with the ideal attributes of PHC as highlighted in the WHO framework across all categories, although Denmark, the Netherlands, and to a lesser extent Kazakhstan, come closer to this model than the others.

### The primary care workforce

#### NHS systems

Across these systems, there is a largely uniform workforce education and composition characterised by multi-professional teams with physicians and nurses as the largest groups forming the core of PHC. These teams demonstrate some degree of skill-mix, task-shifting and integration of new roles for nurses. Academic education is the dominant model for most groups, including specialised training for nurses. While specialisation is mandatory for GPs in most cases, it remains voluntary for other professions. Sector-disaggregated data for relevant PHC groups is publicly available in all or most NHS countries, except in Portugal, facilitating monitoring and planning efforts. Labour market trends point to GPs shortages and more diverse trends for other groups.

#### SHI systems

In established SHI systems, specialised GPs dominate the workforce, although composition varies strongly across countries. The Netherlands stands out for its multi-professional teams including specialised PHC nurses, midwives, physiotherapists, and other providers alongside GPs. Conversely, in Germany and Switzerland, GPs mainly work with medical assistants with task-shifting and skill-mix still in developmental stages. While academic training for GPs is similar across these countries, variations exist for other groups. In the Netherlands, the academisation of a wide range of professional groups is more advanced. For example, the country offers the PHC specialisation of nurses, whereas nurses in Germany and Switzerland lack such opportunities. Sector-disaggregated labour market monitoring is absent across all countries, with only public data available for GPs, and HCWF planning is most comprehensive and advanced in the Netherlands (targeting GPs) and least advanced in Switzerland.

#### Emergent SHI systems

In emergent SHI systems, PHC includes a diverse array of professions, with GPs and nurses serving as major groups. Approaches to task-shifting, team-based care and the integration of new roles vary strongly. Kazakhstan is a forerunner in this regard, while Serbia encompasses a traditional hierarchical model with limited task-shifting. Romania finds itself somewhere in the middle of both. Education and specialisation generally improved, with Kazakhstan seeking to up-skill relevant PHC groups and strengthening inter-professional education, while Romania and Serbia continue to focus on physicians. Comprehensive disaggregated PHC data is lacking across all emergent SHI systems except for physicians, posing challenges for workforce planning and evidence-based decision-making.

#### Trends across healthcare system types

Overall, this analysis reveals strong differences in the workforce composition. While GPs are central to workforce composition in most countries, Serbia and Kazakhstan feature specialist physicians alongside GPs. Despite ongoing efforts to increase the skill-mix and improve education, disparities persist, particularly in SHI systems (established and emergent) where disaggregated data remains scarce, shaping a workforce planning landscape that is biased towards physicians and hampering evidence generation.

### Primary healthcare workforce action

#### NHS systems

Shortage and maldistribution of nurses, physicians, and other relevant PHC groups plague NHS systems, with varying policy approaches across countries. In Denmark, policies focus on structural changes and community-centred approaches supported by bottom-up measures and robust coordination mechanisms. England’s intervention primarily focuses on meso–micro-level organisational (some professional) changes, although with poor implementation and ineffective rollouts for new roles despite increased funding for professional development. Portugal shows overall poor interventions and unclear implementation, although some signs of community-centred efforts are emerging. The evaluation of interventions remains sparse across all countries, with Denmark showing more advanced efforts. Neither the WHO PHC-oriented model nor the Sustainable Development Goals (SDGs) have any relevant impact on policies and interventions in these countries.

#### Established SHI systems

Shortages of GPs and other PHC staff pose similar challenges in established SHI systems, exacerbated by impending retirement cohorts, particularly in Germany and Switzerland and to a lesser degree in the Netherlands (Table 1). Despite this fact, none of the countries are prioritising the primary HCWF in their policy agendas and no systematic responses have been developed. Interventions mainly focus on organisational changes, although these show a limited focus on governance and weak or altogether absent efforts to improve the skill-mix, task-shifting and the development of new roles, except in the Netherlands. Implementation is hampered in all countries by strong corporatism and market forces, compounded by decentralisation in Germany and Switzerland. As in the NHS systems, the WHO PHC-oriented model and the SDGs have little or no impact on policies and interventions.

#### Emergent SHI systems

The emergent SHI systems face similar challenges of workforce shortages and geographical mal-distribution. Serbia and Romania also exhibit a lack of coordination of education and labour markets, high outward-migration rates, and demographic factors all of which do not seem to play a relevant role in Kazakhstan. Policy responses vary strongly between the three countries and are (at least nominally) most advanced in Kazakhstan. Here, the PHC workforce is prioritised, and multi-professional teams are emphasised. Romania has made some efforts in professional education and team-based approaches, while Serbia lags behind with poor policy efforts. Despite some interventions across all countries, only Kazakhstan has set priorities and taken comprehensive sector-specific action, albeit with challenges pertaining to monitoring and implementation. The WHO PHC-oriented model, and to a lesser degree the SDGs, provide some guidance in all countries, but specifically show strong usage in Kazakhstan where the frameworks drive change in PHC workforce development.

#### Trends across healthcare system types

Overall shortages and maldistribution of HCWs are present across all countries. Policy responses and interventions range from governance to organisational, and professional/education measures, although with weaknesses in the implementation of policies and a systematic lack of data and evaluation. The WHO PHC-oriented model and the SDGs only marginally inform policy directions with Kazakhstan standing out for its stronger alignment with these frameworks in comparison to the other countries.

## Discussion

This comparative research provides novel empirical insights that contribute to a deeper understanding of the PHC workforce [30] and prompts critical reflections on the practical implications of the PHC principles put forth in the ‘PHC primer’ [26]. The findings document strong gaps between the idealistic global PHC debate, which seeks standardisation, and the complex realities of diverse PHC systems and workforce conditions in practice. This study reveals an absence of typical PHC workforce teams [19, 28, 47, 48] and a weak community-orientation across our country samples, with the exception of Denmark (Table 2). This illustrates a substantial implementation gap and accentuates the persisting relevance of institutional pre-requisites in shaping PHC provision [12, 31–33]. Available tools and a one-size-fits-all discourse provide little opportunity to systematically address the implementation challenges, as diverse system conditions and governance arrangements remain largely invisible. The same holds true when thinking about potential windows of opportunity for transformative policies.

By advocating for a health system approach and shifting the debate from universal global strategies to the nuanced realities of PHC systems, our research stresses the importance of contextual factors in driving change. The empirical evidence produced in our comparative assessment supports this line of argumentation, revealing that each country has taken some action (Table 4), thereby illuminating transformative policies and related governance capacities. Table 5 provides an overview of the diverse paths through which the global PHC debate may, or may not, intersect with the realities of healthcare systems, considering both strategic and operational dimensions of governance. Importantly, the summary is neither exclusive nor comprehensive but seeks to show broader emergent trends.

**Table 5:** Policies and governance capacities: major trends

System types	Countries	Policies and governance capacities		
		System level	Organisational level	Professional level
NHS systems	Denmark	X	X	X
	Portugal	(X)		X
	UK/England	(X)		X
established SHI systems	Germany		X	(X)
	Netherlands	X	X	X
	Switzerland		X	
emergent SHI systems	Kazakhstan	X	X	X
	Romania	X		(X)
	Serbia	X	(X)	

Source: authors’ own table, based on Tables 2, 3 and 4

Our findings reveal the absence of a coherent pattern pertaining to institutional pre-requisites, PHC workforce conditions, and policy interventions across countries. For instance, the NHS system in Denmark relies on GPs working in independent private businesses contracted by regions, but improved transsectoral coordination and began placing greater emphasis on home care (Table 2). In the SHI system in Netherlands, corporatism (with strong professional actors) and private practices co-exist with complex interventions and more transformative governance, including a wide range of professional groups and stronger public/state control (Table 2). On a negative note, high out-of-pocket payments are characteristic of emergent SHI systems, most pronounced in Kazakhstan [61], but also exist in the Portuguese NHS system [64], and to a lesser degree in the SHI system in Switzerland [69].

The research suggests that isolated policy interventions in workforce governance may not achieve sufficient transformative powers in the PHC system at large. For instance, while initiatives like the introduction of new nursing roles in Portugal [74] and incentives for GPs in Germany [75] show promise, they may fall short of achieving systemic change. On the other hand, multi-level governance actions and policy coordination together with community-centred approaches embody stronger transformative potential that may bring the PHC workforce closer to the global model [7, 26]. However, it is precisely these comprehensive policy approaches that often encounter hurdles in their implementation with many countries lacking effective transsectoral and multi-professional coordination mechanisms needed to address the complex challenges facing PHC provision and workforce crisis.

These findings have important implications for the PHC debate and the development of tools and strategies to effectively respond to the ‘layered crisis’ [30] of the PHC workforce. They call for greater attention to healthcare systems and implementation, addressing existing governance gaps across different levels.

### Limitations

This study has a few important limitations that should be considered. First, we employ a qualitative explorative approach, which helps identify challenges and opportunities while promoting context-sensitivity. However, this method does not permit drawing quantitative conclusions about the PHC workforce. Second, our rapid assessment relies on expert information and secondary sources, and the selected countries are not exhaustive. Although we aimed for diversity by including countries with varied healthcare systems and HCWF compositions (Table 1), it is possible that other relevant items may have been overlooked. Third, this study does not specifically consider gender disparities [76–78] and other inequalities within the PHC workforce, such as the role of migrant HCWs [17, 79–81]. These are important considerations, but data limitations and the scope of our research precluded their examination. Fourth, complexity of PHC systems and the HCWF make interventions and policy implementation at the interface highly complex processes with eventually diverse outcomes. We identified major dimensions of the ‘layered crisis’ [30] and some governance capacities that might support transformations. However, these may not provide a comprehensive picture of all opportunities as there may be additional dimensions of the implementation gap, as well as of the transformative capacities, that our research did not address. Finally, it is important to recognise that this study serves as a pilot initiative, highlighting the need for and benefits of more extensive comparative research, particularly in identifying more comprehensive governance mechanisms. Future studies should build upon our methodological approach in order to research the specific aspects of PHC governance, workforce dynamics, and inequalities more comprehensively.

## Conclusions

Our qualitative comparative assessment lays the groundwork for more targeted research questions on PHC workforce governance, specifically to address the layered complexities across diverse health systems. It argues that integrating the debates surrounding PHC and the HCWF crisis while placing greater emphasis on the role of implementation is paramount. By employing a health system and governance approach along with a rapid comparative assessment using qualitative methods and country case studies, we were able to track opportunities for more transformational policies within the broader health system context. Aligning PHC and HCWF discourse and disentangling the layers of the workforce crisis may help move the debate forward and build governance capacities to improve resilience in both areas. Our conceptual framework and empirical findings support the development of evidence-based and context-sensitive policy recommendations.

## Key recommendations

- Recognise the crucial role of the healthcare workforce as the backbone of PHC systems and advocate for coordinated multi-level governance action to support their effectiveness.
- Shift the PHC debate from idealistic attributes to actionable implementation strategies, emphasising the significance of policy dynamics, political contexts, and diverse stakeholder interests.
- Understand the various existing PHC-oriented models and their dynamics to determine the necessary quantity, competencies, and composition of HCWs and how they can be governed effectively to implement PHC.
- Establish a health system and governance approach together with qualitative comparative PHC workforce studies to identify and develop transformational policies within specific contextual settings.
- Prioritise investments in PHC-disaggregated workforce data and monitoring mechanisms to improve evidence-based policymaking and strategic workforce planning.
- Strengthen knowledge exchange and collaboration among international health organisations, governments, and stakeholders to understand and effectively use diverse transformative policies and governance capacities.

## Supplementary Information

Additional file 1: References for the nine country cases

Additional file 2: Matrix

## Data Availability

All data generated and analysed during this study are included in this published article and supplementary information files.

## Abbreviations

APN: Advanced Practice Nurse
EU: European Union
HCW: Healthcare workers
HCWF: Healthcare workforce
NHS: National health service
PHC: Primary healthcare
SDGs: Sustainable Development Goals
SHI: Social health insurance
UHC: Universal health coverage
UK: United Kingdom
WHO: World Health Organisation
