# Supplementary material for: Tackling the primary healthcare workforce crisis: time to talk about health systems and governance—a comparative assessment of nine countries in the WHO European region
Source: Hum Resour Health. 2024 Dec 31;22:83. doi: 10.1186/s12960-024-00965-2 (PMC11686866; doi:10.1186/s12960-024-00965-2)
Supplement: Supplementary file 2 — Additional file 2: Matrix [file 12960_2024_965_MOESM2_ESM.docx]

**Supplementary material 2**

**Matrix**

| Country |  |
| --- | --- |
| PHC sector overview | |
| - Provision |  |
| - Finance |  |
| - Governance |  |
| PHC healthcare workforce | |
| - Main professions/occupations |  |
| - Education |  |
| - Labour market figures |  |
| - HCWF data, monitoring, planning |  |
| - HCWF governance |  |
| PHC workforce policy | |
| - Problems/challenges |  |
| - Policy |  |
| - Interventions/policy implementation |  |
| Global goals/frameworks | |
| - Role of the WHO PHC concept |  |
| - Role of the SDGs |  |

Background information

References, documents
